# Trends in Pediatric Complicated Pneumonia in an Ontario Local Health Integration Network

**DOI:** 10.3390/children5030036

**Published:** 2018-03-03

**Authors:** Tahereh Haji, Adam Byrne, Tom Kovesi

**Affiliations:** 1 College of Medicine, University of Saskatchewan, Saskatoon, SK S7N 5E5, Canada; tahereh.haji@usask.ca; 2 Department of Pediatrics, Children’s Hospital of Eastern Ontario, 401 Smyth Rd., Ottawa, ON K1H 8L1, Canada; adbyrne@cheo.on.ca

**Keywords:** pneumonia, empyema, pleural effusion, child pneumococcal vaccines, pneumococcal infections

## Abstract

Following the introduction of 7-valent pneumococcal vaccine (PCV7), while overall rates of invasive pneumococcal disease and pneumococcal pneumonia in children declined, rates of empyema increased. We examined changes in the incidence of hospitalization for pediatric complicated pneumonia (PCOMP) in Eastern Ontario, Canada, particularly since the introduction of the 13-valent vaccine (PCV13). A retrospective chart review was carried out evaluating previously healthy children admitted with PCOMP, which included empyema, parapneumonic effusion, necrotizing pneumonia, and lung abscess between 2002 and 2015. Three-hundred seventy-one children were included. Subjects had a median age of four years, and 188/370 (50.8%) required a chest tube. Admission rates changed markedly during this time period. The number of admissions per year rose most sharply between 2009 and 2012, corresponding to the period following introduction of PCV7 and then the occurrence of pandemic influenza A (H1N1). In children who likely received PCV13, the incidence of PCOMP returned to approximately pre-PCV7 levels. In contrast, rates of PCOMP in older children (who would not have received PCV13) remained elevated during the post-PCV13 time period. While rates of PCOMP, particularly in older children, remain elevated following the introduction of PCV13, this might be expected to resolve with more widespread vaccine coverage with PCV13 and herd immunity.

## 1. Introduction

Pediatric complicated pneumonia (PCOMP) is defined as pneumonia complicated by parapneumonic effusion (a transudative pleural effusion associated with pneumonia); empyema (purulent fluid in the pleural space); necrotizing pneumonia (liquefaction and cavitation of consolidated lung tissue associated with empyema); or lung abscess (necrosis of lung parenchyma producing one or occasionally several large thick-walled cavities) [1,2,3]. *Streptococcus pneumoniae*, the most common cause of bacterial pneumonia in children, is also the most common bacteria causing PCOMP, though not lung abscess. Other organisms can cause these infections in children, as well, including other Streptococcal organisms, *Staphylococcus aureus*, and *Enterobacteriaceae* [4].

Reductions in hospitalization rates for all-cause pneumonia and invasive pneumococcal disease have been widely reported since the introduction of the 7-valent pneumococcal vaccine (PCV7) [5], and a decline in the incidence of these conditions possibly started even earlier [6]. Despite this, an increase in hospitalizations for empyema and other forms of PCOMP has been described in disparate geographic regions [7,8,9,10,11]. For example, in the intermountain region of the Western United States, Byington et al., observed that after the introduction of PCV7, while the rate of invasive pneumococcal disease decreased by 27%, the proportion of cases complicated by empyema rose from 16 to 30% [7]. Similarly, in Australia, while the overall rate of hospitalization due to pneumonia decreased by 22%, the rate of hospitalization for empyema increased 35% in the post-PCV7 period [12]. The increase in empyema due to pneumococcus appears to have been largely driven by serotype replacement in the community with non-PCV7 serotypes–particularly serotype 19A [9,13], 1 [14,15], and 3 [13]. In contrast, a province-wide study from Quebec, Canada found that while rates of pediatric empyema increased from 0.23/100,000 in 1990 to 4.01/100,000 person-years in 2007, the rise began before the introduction of PCV7 [16]. Other bacteria may also be contributing towards an increasing incidence of PCOMP, most notably, methicillin-resistant *Staphylococcus aureus* (MRSA) in the United States [1,13,17]. It is also conceivable that population changes in the respiratory microbiome have influenced the incidence of PCOMP [18]. As bacterial pneumonia typically follows a viral respiratory tract infection in children, rates of both pneumonia and PCOMP vary considerably from year to year, depending on the severity of winter outbreaks of respiratory viruses, such as influenza virus and Respiratory Syncytial Virus [5].

In Ontario, public funding for PCV7 commenced in 2005. Public funding of PCV7 was briefly superseded for one year in the province of Ontario by a 10-valent conjugate pneumococcal vaccine (PCV10) in 2009, and then by a 13-valent vaccine conjugate pneumococcal vaccine (PCV13) in late 2010 [19]. It includes serotype 19A in addition to five additional serotypes (1, 3, 5, 6A, and 7F). Since the introduction of PCV13, overall rates of invasive pneumococcal disease [20] and pneumococcal pneumonia [21,22] have continued to decline. However, relatively few studies have evaluated the effect of PCV13 on PCOMP.

The aim of the study is to describe changes in rates of PCOMP hospitalizations spanning the pre-PCV7 to the post-PCV13 periods in a geographically circumscribed area. We particularly wished to examine overall trends before the introduction of PCV7 and after the introduction of PCV13.

The Children’s Hospital of Eastern Ontario (CHEO) in Ottawa, Ontario, Canada, is the only tertiary care pediatric hospital in our health region in Eastern Ontario (the Champlain Local Health Integration Network (CLHIN)), while also acting as a referral center for Western Quebec and part of Nunavut. Essentially all children with PCOMP in the CLHIN are admitted to CHEO. Previous polymerase chain reaction (PCR) studies based on a large convenience sample of children admitted to CHEO with pneumonia complicated by effusion showed that 62% were caused by *S. pneumoniae*, while 16% were caused by Group A *Streptococcus*. While MRSA has been identified as an important and emerging cause of PCOMP, it is uncommon in our region [17,23].

## 2. Materials and Methods

We performed a retrospective review of cases of PCOMP in previously healthy children admitted to CHEO between 2002 and 2015. We defined PCOMP as the presence of parapneumonic effusion, empyema, lung abscess, or necrotizing pneumonia. Cases were identified by a computerized search of hospital medical records using the International Statistical Classification of Diseases and Related Health Problems Canadian Coding Standards (ICD-10-CA codes), including detailed codes (Table S1). Coding changed from the International Classification of Diseases, Ninth Revision, Clinical Modification (ICD-9-CM) to ICD-10-CA in April 2002, but we verified with the CHEO Health Information Decision Support Team that this did not impact our search terms (Supplementary Materials Table S1) as annual discharges at CHEO are calculated based on the fiscal year (1 April to 31 March) rather than the calendar year. One minor change in codes took place since the implementation of ICD-10-CA, but we felt this was unlikely to affect our case ascertainment (see Supplementary Materials Table S1). Cases identified by the computerized search underwent chart review. Data from the chart review was entered into a secure database (REDCAP™ Vanderbilt University, Nashville, TN, USA). The chart review focused on the discharge summary, but, when necessary, progress notes and laboratory data were examined. We recorded demographics, date and length of admission, the type of complication, type of effusion, lung lobe involvement, microbiologic testing, imaging reports, immunization status (when available) and management (chest tube and/or video-assisted thoracoscopic surgery (VATS)). If a patient had two complications during the same admission, the one most significant to their care was used. Whether an effusion was classified as a parapneumonic effusion or an empyema was based on the diagnosis provided in the medical record. The accuracy of documentation of vaccination status in the medical history was not verified with either the primary care physician or the provincial health record.

We defined “previously healthy” children as children admitted with a community-acquired pneumonia who did not have a condition that predisposed them to aspiration (such as neurodevelopmental disorders with swallowing dysfunction, laryngeal cleft, tracheoesophageal fistula, and/or gastroesophageal reflux) and who did not have a history of chronic respiratory disease (excluding asthma, apart from those on high-dose corticosteroid therapy); chronic cardiac disease; cirrhosis of the liver; chronic renal disease or nephrotic syndrome; diabetes mellitus; asplenia, splenic dysfunction, sickle-cell disease and other sickle-cell hemoglobinopathies; chronic cerebrospinal fluid leak; primary immune deficiency; and/or human immunodeficiency virus (HIV) infections and other conditions associated with immunosuppression (malignancies, long-term systemic corticosteroids and other immunosuppressive therapy, or solid organ transplant recipients) documented in the CHEO medical record. Children with these conditions were excluded from the study. Children transferred from secondary-level hospitals with community-acquired pneumonia because of the development of PCOMP were not excluded.

The data was exported from REDCAP™ to Microsoft Excel 2013 (Microsoft, Redmond, WA, USA), recoded, and then exported to IBM SPSS Statistics version 24 for analysis (IBM, Armonk, NY, USA). We found that sufficient data to categorize effusions as parapneumonic or empyema using Light’s criteria [24] was infrequently available in the hospital records. In addition, we were concerned that increasing use of thoracic ultrasound over time likely increased the likelihood that effusions were classified as empyemas. Therefore, parapneumonic effusions and empyemas were analyzed both separately and together. Moreover, we felt that pleural fluid collections requiring placement of a chest tube were much more likely to be clinically significant, and the incidence of tube thoracostomy was also analyzed.

As a denominator for hospitalization rates, the number of children living in the Champlain Local Health Integration Network (CLHIN) in each of the study years was obtained from the CLHIN (information available on request). This data was not available for 2014 and 2015. Extrapolated numbers of children for each of these years were determined using a linear regression equation generated by the available data from the previous years; the number of children living in the CLHIN dropped slightly, but significantly, during the study time period (*p* < 0.001) (data available on request). The number of children in each age group was estimated by dividing the total number of children under 18 years living in the CLHIN in each year by 18, assuming that the age distribution of children in the CLHIN is approximately even and the age distribution did not substantively change during the study time period. We confirmed that the proportion of children in different age groups did not change substantively during the study period: based on Canadian census data, the proportion of children living in the city of Ottawa who were between 0–4 years of age was 5.76% in 2001 [25], 5.46% in 2006 [26], and 5.56% in 2011 [27]; the proportion between 5–14 years were 13.12%, 12.11%, and 11.26%, and between 15–19 years were 6.39%, 6.72%, and 6.60%, respectively. In addition, using national census data for the city of Ottawa, we confirmed that the number of children of each year of age (between 0 and 19) was relatively even, varying between only 8917 and 10,157 in 2011, 8875 and 10,919 in 2006, and 9828 and 11,661 in 2011. Children living in the catchment area were assumed to be healthy, as the proportion of children who have underlying medical conditions was unavailable to us.

PCV7 was incorporated into the Ontario regular vaccine schedule in 2005, and PCV13 became widely utilized in Ontario in 2011 [19]. The study time period was, therefore, divided into three intervals:

- 2002–2004 was considered the pre-PCV7 period;
- 2005–2011 was considered the post-PCV7/pre-PCV13 period; and
- 2012–2015 was considered the post-PCV13 period.

While PCV10 briefly replaced PCV7 in the provincially-funded program in 2009, it was replaced, in turn, by PCV13 in late 2010; given that this vaccine was used for only a brief, one-year time interval, this period was not analyzed separately [19]. Children were considered to have received PCV13 if they were under 2 years of age in 2013, under 3 years of age in 2014, or under 4 years of age in 2015.

We adopted a null hypothesis that the frequency of PCOMP hospitalization did not change between the pre-PCV7 period and post-PCV13 period. Visual inspection of admission rates suggested that peak admission rates corresponded to the outbreak of pandemic influenza A (H1N1) in 2009–2010 [28] and, presumably, the period of maximal *S. pneumoniae* serotype replacement with 19A, between 2008 and 2012. Given the likely confounding of PCOMP admission rates by pandemic influenza A (H1N1), and the well-recognized increase in PCOMP after the introduction of PCV7, we felt the rates of PCOMP hospitalization in the post-PCV13 era would be best evaluated by comparing these rates to the pre-PCV7 period. Moreover, assuming PCV13 would markedly reduce PCV13 serotypes in vaccinated (younger) children [21], we felt it was also important to evaluate whether this produced “herd protection” and control of PCOMP throughout the pediatric age range, by potentially reducing population-wide nasopharyngeal colonization of PCV13 serotypes [29].

While CHEO also provides tertiary-level pediatric care for children from Western Quebec and Eastern Nunavut, these patients were not analyzed because of differing times of incorporation of PCV into the immunization schedule, and widely-disparate socio-economic conditions, respectively. In addition, the number of children admitted from these regions with PCOMP was relatively small.

Data were analyzed using IBM SPSS Statistics. Trends in continuous variables were analyzed using linear regression or one-way analysis of variance (ANOVA). The number of children living in the CLHIN who were not admitted to CHEO with PCOMP in each year were introduced into SPSS using the WEIGHT command. Proportions were compared using chi-square or Fisher exact tests (depending on the expected cell size). A probability (*p*) value < 0.05 was considered statistically significant.

Approval to conduct this retrospective, chart review-based study was obtained from the CHEO Research Ethics Board, Children’s Hospital of Eastern Ontario Research Institute, Ottawa, ON Canada (REB Protocol No. 14/83X).

## 3. Results

### 3.1. All Children in the Champlain Local Health Integration Network

During the study period of 1 January 2002 and 31 December 2015, approximately 202,358 to 370,408 children lived in the CLHIN. During the study period, 492 children with PCOMP were admitted to CHEO. Thirty children were excluded because of pre-existing co-morbidities, and 91 were excluded because they lived outside the CLHIN, leaving 371 eligible for inclusion in the study. Their median age was 4 years (range 0.25–17 years). Of the study population, 47.2% (175/371) were male. Age at the time of admission did not change significantly during the study period (one-way ANOVA, F (3357) = 1.13, *p* = 0.34). The median length of stay was 10.0 days (range 1–73 days); 50.8% (188/370) required a chest tube, 13.5% (50/370) had chest tube placement during VATS, while the remainder had chest tube placement performed by either general surgery or interventional radiology. No patient had a lobectomy or drainage of a lung abscess. There was no significant change in the proportion of patients receiving a chest tube over time (*p* = 0.089). The proportion of children undergoing VATS varied significantly during the course of the study period (*p* < 0.001) and peaked in 2008; no patient underwent VATS in 2014 or 2015. The most commonly affected lobes were the left lower lobe (188/371, 50.7%) and right lower lobe (151/371, 40.7%) (Table 1). Immunization status was reported to be “up-to-date” in 87.3% (324/371) patients, not up-to-date in 0.5% of patients (2/371), and unknown in 12.1% of patients (45/371).

**Figure 1:**
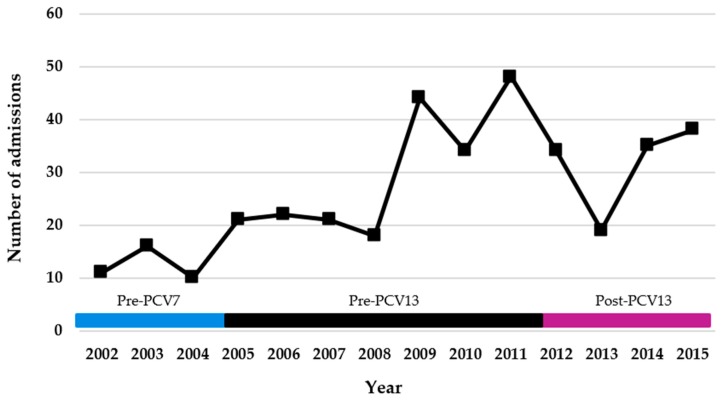
Total number of admissions per year for complicated pneumonia, CLHIN (all age groups). Periods: pre-7-valent pneumococcal vaccine (PCV7) 2002–2004 (37 cases); post-PCV7 and pre-PCV13 2005–2011 (208 cases); post-PCV13 2012–2015 (126 cases).

The number of children residing in the CLHIN admitted with PCOMP ranged from 11 to 48 cases per year. The number of cases tended to rise between 2002 and 2008, peaked between 2009 and 2012, and then tended to drop slightly. There were significant changes in the number of admissions per year, over the study period (*p* < 0.001) (Table 2, Figure 1). Similar trends were noted for admissions for empyema (*p* = 0.045), parapneumonic effusion (*p* < 0.001), all effusions (*p* < 0.001), and the requirement for a chest tube (*p* ≤ 0.001), but not for necrotizing pneumonia (*p* = 0.17) or lung abscess (*p* = 0.52) (Table 2). Very similar temporal trends were observed for children with PCOMP admitted from Western Quebec, although these patients were not statistically evaluated due to the small sample size (Figure S1).

Among children admitted during the pre-PCV7 or post-PCV13 time periods, 9/38 (23.7%) and 14/126 (11%) had positive cultures, obtained from blood, pleural fluid, and/or broncho-alveolar lavage specimens. *S. pneumoniae* and Group A *Streptococcus* accounted for 43.5% and 26.1% of all positive cultures, respectively (Table 3). During the pre-PCV7 period, cultures were positive for *S. pneumoniae* in 5 (55.6%) and Group A *Streptococcus* in 3 children (33.3%). During the post-PCV13 period, cultures were positive for these organisms in five (35.7%) and three children (28.6%), respectively. During these two periods, blood cultures were most often positive for *S. pneumoniae* (7/11 patients with positive blood cultures, 63.4%). Pleural cultures were most often positive for Group A *Streptococcus* (5/12 patients with positive pleural cultures, 41.7%).

### 3.2. Children in the CLHIN during the Pre-PCV7 Period and Children Who Likely Received PCV13 in the Post-PCV13 Period

**Figure 2:**
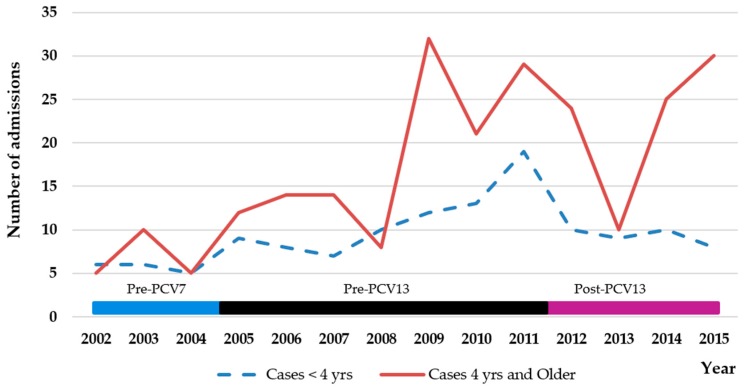
Total number of admissions per year for complicated pneumonia, CLHIN, by age group. Periods: pre-PCV7 2002–2004 (17 cases < 4 years, 20 cases > 4 years); post-PCV7 and pre-PCV13 2005–2011 (78 cases < 4 years, 130 cases > 4 years); post-PCV13 2012–2015 (37 cases < 4 years, 89 cases > 4 years).

**Figure 3:**
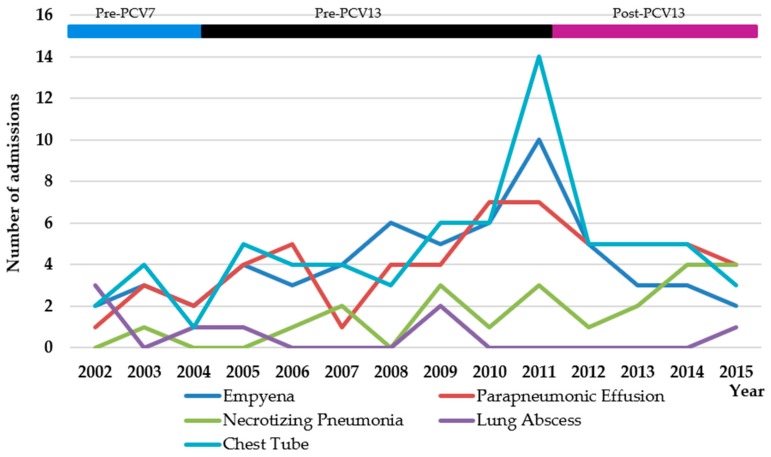
Number of children under four years of age living with CLHIN with complications of pneumonia or placement of a chest tube, by year.

Over the course of the entire study period (2002–2015), there were no significant changes in admission rates for PCOMP (*p* = 0.16) (Table 4, Figure 2). Similarly, there were no significant changes in admission rates for the various types of PCOMP, although a significant increase in the requirement for a chest tube was observed (*p* = 0.024) (Table 4, Figure 3). The incidence of admission for parapneumonic effusion (*p* = 0.048), and all effusions (*p* = 0.046) was significantly higher in children under four years of age in the post-PCV13 period than during the pre-PCV13 period. However, admission rates for PCOMP (*p* = 0.072), empyema (*p* = 0.44), necrotizing pneumonia (*p* = 0.14), lung abscess (*p* = 0.10), and the requirement for a chest tube (*p* = 0.12) were not significantly different (Table 4, Figure 3).

### 3.3. Older Children in the CLHIN Who Would Be Unlikely to Have Received PCV13 in the Post-PCV13 Period

**Figure 4:**
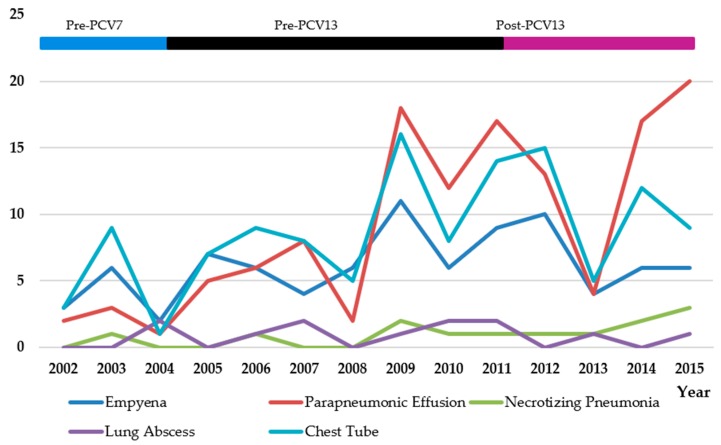
Number of CLHIN children four years of age or older with complications of pneumonia or placement of a chest tube, by year.

Over the course of the entire study period (2002–2015), admission rates for PCOMP increased significantly (*p* < 0.001) (Table 4, Figure 2). Specifically, significant increases in admission rates were observed for parapneumonic effusion (*p* < 0.001), all effusions (*p* < 0.001), and the need for a chest tube placement (*p* = 0.005). No significant changes were observed in admission rates for empyema (*p* = 0.39), lung abscess (*p* = 0.58), or necrotizing pneumonia (*p* = 0.53). Compared to the pre-PCV7 period, admission rates in the post-PCV13 period were significantly increased for PCOMP (*p* < 0.001), parapneumonic effusion (*p* < 0.001), all effusions (*p* < 0.001), and need for placement of a chest tube (*p* = 0.003), but not for empyema (*p* = 0.089), lung abscess (*p* = 0.80), or necrotizing pneumonia (*p* = 0.076) (Table 5, Figure 4).

### 3.4. Sensitivity Analyses

We performed several sensitivity analyses to further validate our findings. We analyzed PCOMP relative to all-cause admissions to CHEO during the study time period. The total number of admissions to CHEO per year ranged from 5879 to 6716. Over the course of the entire study period (2002–2015), PCOMP hospitalizations changed significantly for the entire study population (*p* < 0.001) and for children 4 years of age and older (*p* < 0.001). In contrast, there were no overall significant trends for children under four years of age (*p* = 0.39). Rates of PCOMP were significantly higher in the post-PCV13 period than in the pre-PCV13 period for the entire study population (*p* < 0.001) and children four years of age and higher (*p* < 0.001), but not in children under four years of age (*p* = 0.22).

In addition, we compared admission rates for PCOMP to hospitalizations at CHEO for an unrelated diagnosis during this time period—urinary tract infections (UTI). Admissions for UTI ranged from 73 to 164/year. Due to the relatively low number of UTI admissions per year, PCOMP admissions were compared to UTI admissions only for the entire study population. Relative to UTIs, PCOMP admission rates changed significantly during the study period (*p* < 0.001). Compared to UTI, rates of PCOMP were significantly higher in the post-PCV13 period than in the pre-PCV13 period (*p* = 0.001).

## 4. Discussion

While rates of invasive pneumococcal disease and pneumococcal pneumonia have been observed to continue falling following the implementation of routine immunization with PCV13, few studies have evaluated the impact of PCV13 on empyema and other forms of PCOMP. In this study, we observed that the incidence of PCOMP in Ontario children residing in the CLHIN appeared to undergo considerable variation between 2002 and 2015. Compared to the pre-vaccination period, the number of admissions for PCOMP and the number of patients receiving tube thoracostomy rose notably between 2009 and 2012. These increases could have been due to a combination of serotype replacement following introduction of the PCV7 vaccine in 2005 and, potentially, the occurrence of pandemic H1N1 influenza A [30]. As rates of PCOMP remained higher between 2011 and 2012, after pandemic influenza had abated, persistently higher rates of PCOMP during this time interval could have been due to several potential factors, including continued serotype replacement by non-PCV7 serotypes associated with empyema, changes in pneumococcal virulence, an increased prevalence of other causal bacteria, and changes in practice. However, rates of PCOMP tended to decrease back towards pre-PCV levels in children who likely received PCV13, suggesting that PCV13 both prevents invasive pneumococcal disease in general, and can reverse the observed recent trend in rising rates of PCOMP. Consistent with this finding, a recent study in Greece determined that since implementation of PCV13, no cases of empyema caused by 19A or 7F were observed [31]. Although overall rates of PCOMP following the adoption of PCV13 remained elevated in the post-PCV13 era, this increase was considerably more substantial in children four years of age and older—a group that has not yet received PCV13. These findings could be due to the possibility that the reduction in PCV13 serotypes in young children vaccinated with PCV13 has not yet led to herd immunity in older individuals not vaccinated with PCV13. Alternatively, given that only a minority of children with PCOMP had positive cultures for *S. pneumoniae*, it is possible that trends in the incidence of this condition are related to infection with other organisms, such as Group A *Streptococcus* or *Mycoplasma pneumoniae* [32]. Changes in the microbiome in the community [14] could also influence the incidence of this disease, as could differential development of immunity to the various serotypes contained in the PCV vaccines [33]. Trück et al., reported that children who had received PCV13 were less likely to maintain high titers of antibodies to serotypes 1 and 3 compared to other PCV13 serotypes [34]. It is possible that the virulence of *S. pneumoniae* and other bacteria causing PCOMP had changed, as there is evidence that the incidence of PCOMP began to rise before PCV7 was introduced [16]. The incidence of lung abscesses and necrotizing pneumonia changed little over time, suggesting that the microbiology, epidemiology, and/or pathogenesis of these conditions differs from pneumonia complicated by effusion.

Our study had a number of strengths. It took place in a quite strictly defined catchment area, so it was unlikely that serious cases were missed. By reviewing charts directly, rather than using large healthcare databases, the validity of diagnoses could be examined, and diagnoses other than empyema could be excluded. MRSA PCOMP appears to be uncommon in our region, and would therefore be unlikely to contribute significantly to changes in the incidence of PCOMP in the CLHIN [23]. Group A *Streptococcus* has been reported to be an important cause of severe pneumonia in the pediatric population [35], and this was reflected by our observations on the results of pleural culture. Deceuninck et al. also found that the organisms most commonly isolated in children with empyema were *S. pneumoniae* and Group A *Streptococcus*, which were isolated in 42% and 30%, respectively, of positive cultures [16]. Our findings were supported by sensitivity analyses substituting total hospital admissions or admission for UTI for the at-risk population within the CLHIN.

Our study also had a number of limitations. As in all retrospective reviews, cases may have been missed due to diagnostic coding misclassification. Some children included in the study may have had underlying conditions not recorded in the medical record, and an unknown number of unaffected children had underlying conditions. Deceuninck et al. reported that among children with empyema in Quebec, the commonest underlying condition was asthma, which was seen in 21% of cases; 17.8% presented with other medical conditions [16]. The proportion of children in the various age groups was assumed and presumed to be constant during the study time period. The sample size was smaller than previous studies evaluating entire states or provinces. Moreover, the number of children under four years of age was relatively small, raising the possibility of a type II error when evaluating vaccinated or vaccine-eligible children. While the change in ICD codes did not affect case ascertainment for our study, cases were identified by fiscal year and the at-risk population for each year was identified by calendar year. Given the small changes in the size of the pediatric population in the CLHIN from year to year, this was unlikely to have affected our findings. The size of effusions could not be consistently determined by the medical record, and small, non-clinically significant pleural effusions may have been included. Similarly, absence of sufficient information to apply Light’s criteria led to uncertainty whether individual cases likely represented empyema or parapneumonic effusion. Moreover, as reported elsewhere, during the study period, ultrasound became standard-of-care for evaluation of pleural effusion associated with pneumonia, both to diagnose empyema and to plan chest tube placement [16]. As thoracic ultrasound became more widely utilized in the evaluation of pleural effusions, the proportion of effusions with reported septations has increased, leading clinicians to diagnose empyema more readily. The issues of classification of type of effusion were at least partly circumvented by combining both diagnoses in the analyses, and by analyzing the incidence of chest tube placement, which likely represents more serious and clinically-important cases. During the study time period, there were also significant changes in the diagnosis and management of complicated pneumonia at CHEO. Tube thoracostomy became shared between Pediatric General Surgery and Interventional Radiology. The use of VATS increased up until 2008, then declined as new evidence became available suggesting that intrapleural installation of fibrinolytics had similar efficacy and less morbidity [36]. Following a clinical concern over a substantial rise in pneumonia-associated pleural effusions in 2009, we attempted to standardize management by establishing a protocol requiring rapid consultation with departments of respirology, infectious diseases, and general surgery for all new cases of medium-sized or large pneumonia-associated effusions. It is possible that the use of chest tubes was influenced by the establishment of our hospital’s pleural effusion protocol. Furthermore, the lower morbidity associated with soft, pigtail catheters may have influenced the decision to drain effusions. However, as chest tubes are invasive, usually require an anesthetic in children, and even soft catheters are associated with significant pain, clinicians are selective in placing chest tubes unless the effusion is either reasonably large, leading to symptoms, or both. These changes likely influenced the frequency of diagnosis of empyema versus parapneumonic effusion and likely influenced length of stay and use of invasive interventions, but were unlikely to have influenced overall trends in the observed incidence of PCOMP. Vaccination in eligible patients [19] could not be reliably evaluated, as immunization records are not necessarily inspected during admission to hospital. While our observations may reflect, at least in part, the effects of the provincial PCV vaccination program, it is likely that an unknown number of patients, both hospitalized and non-hospitalized, had not been vaccinated. Moreover, given that PCV vaccination is protective against pneumococcal pneumonia, it is possible that children admitted with PCOMP were less likely to have been immunized. Admission rates for pediatric pneumonia vary considerably from year to year, presumably due at least in part to respiratory viral outbreaks, and our observations could be caused by confounding by non-pneumococcal infections—particularly concurrent viral infections [5]. Apart from a temporal relationship between pandemic influenza A (H1N1), we did not account for viral infection epidemics. Data on viral co-infection, including pandemic influenza A (H1N1), were not recorded during our chart reviews, so we are unfortunately unable to more precisely evaluate the relationship between viral infections, including pandemic influenza A, and POCOMP in the study population. The effects of herd immunity are undoubtedly complex, and likely have a bidirectional influence on the incidence of bacterial pneumonia in both children and adults [37]. We did not consider the effects of PCV10 vaccination, though, as indicated, it was only funded provincially for a single year. PCV has been shown to have considerable effectiveness, based on the vaccine type and pneumococcal serotype, including PCV10, which appears to have provided cross-protection against serotype 19A [37]. Bacterial etiology could only be established in a minority of cases, therefore, definitive conclusions as to causality cannot be certain. In patients undergoing thoracentesis, pleural cultures were often negative, as has been reported previously [1]. More consistent use of PCR techniques in the future may allow bacterial diagnosis in a larger proportion of complicated pneumonia cases. We did not have access to data on trends in invasive pneumococcal disease or overall all-cause pneumonia in the CLHIN. We did not have data on pneumococcal serotype carriage, or changes in serotype carriage, in children in the CLHIN. Similarly, serotypes were not consistently available in culture-positive cases. As a result, the relationship between changes in rates of PCOMP admissions and serotype replacement must be viewed as speculative.

## 5. Conclusions

Rates of PCOMP in children in Eastern Ontario began rising in the first decade of this century. Rates of PCOMP in younger, vaccinated patients in our region have since decreased. This appeared to occur contemporaneously with introduction of the PCV13 provincial vaccination program. Rates of PCOMP in older children in CLHIN remain elevated. It is possible that this will abate through a combination of herd immunity and a higher proportion of Ontarians being vaccinated with PCV13. The significant variation in the incidence of PCOMP highlights the need for ongoing surveillance, ideally using molecular diagnosis [38], as both vaccination strategies and the ecology of bacteria in the community continue to evolve. Future studies comparing rates of PCOMP with historical data will also need to consider changes in diagnostic techniques.

## Figures and Tables

**Table 1:** Lobes involved in children (*n* = 371) living in the Champlain Local Health Integration Network (CLHIN) with pediatric complicated pneumonia (PCOMP) (53.4% of the children had one lobe affected, 35.8% had two lobes involved, 10.0% had 3 lobes involved, 0.5% had four lobes involved, and 0.3% had five involved).

Lobe Involved	Number of Children	% of Children
Right Lower Lobe	151	40.7
Right Middle Lobe	113	30.5
Right Upper Lobe	77	20.8
Left Lower Lobe	188	50.7
Left Upper Lobe	59	15.9

**Table 2:** Incidence of PCOMP and chest tube placements per 100,000 children, for children of all ages living in the CLHIN (by year).

Year	Period	At-Risk Population	Empyema	Parapneumonic Effusion	All Effusions	Chest Tube	Lung Abscess	Necrotizing Pneumonia	Total PCOMP Cases
2002	1	279,785	1.787	1.072	2.859	1.787	1.072	0.000	3.932
2003	1	279,120	3.224	2.150	5.374	4.657	0.000	0.358	5.732
2004	1	277,546	1.441	1.081	2.522	0.721	1.081	0.000	3.603
2005	2	274,774	4.003	3.275	7.278	4.367	0.364	0.000	7.643
2006	2	272,684	3.301	4.034	7.334	4.767	0.367	0.367	8.068
2007	2	272,320	2.938	3.305	6.243	4.407	0.734	0.734	7.712
2008	2	273,148	4.393	2.197	6.590	2.929	0.000	0.000	6.590
2009	2	273,284	5.855	8.050	13.905	8.050	1.098	1.098	16.100
2010	2	273,655	4.385	6.943	11.328	5.116	0.731	0.365	12.424
2011	2	273,457	6.948	8.777	15.725	10.239	0.731	1.097	17.553
2012	3	271,952	5.516	6.619	12.134	7.354	0.000	0.368	12.502
2013	3	270,483	2.588	3.327	5.915	3.697	0.370	0.739	7.024
2014	3	269,821	3.336	8.154	11.489	6.300	0.000	1.482	12.972
2015	3	269,127	2.973	8.918	11.890	4.459	0.743	1.486	14.120
*X*^2^ for overall trend ^1^			22.72	63.16	69.91	44.67	12.05	17.82	74.29
*p* for overall trend			0.045	<0.001	<0.001	<0.001	0.52	0.17	<0.001
*X*^2^ for pre-PCV-7 vs. post-PCV-13 ^2^			3.36	30.07	29.20	10.79	1.95	6.07	29.00
*p* for pre-PCV-7 vs. post-PCV-13			0.083	<0.001	<0.001	0.001	0.16	0.014	<0.001

Periods: (1): Pre-PCV7; (2): Post-PCV7 and pre-PCV13; (3): post-PCV13; ^1^ Degrees of freedom for chi-square with multiple groups = 13; ^2^ Degrees of freedom for chi-square = 1.

**Table 3:** Number of bacterial isolates from blood or pleural fluid for children living in the CLHIN with PCOMP in the 2002–2004 (pre-PCV7) and 2012–2015 (post-PCV13) periods.

	Pre-PCV7			Post-PCV13		
Organism	Blood (% of Positive Blood Cultures)	Pleural Fluid (% of Positive Pleural Cultures)	% of All Blood or Pleural Fluid Isolates	Blood (% of Positive Blood Cultures)	Pleural (% of Positive Pleural Cultures)	% of All Positive Blood or Pleural Fluid Isolates
*S. pneumoniae*	4 (100)	1 (20)	62.5	3 (42.9) ^4,6^	2 (28.6)	35.7
Group A *Streptococus*	0 (0)	3 (60)	37.5	1 (14.3)	2 (28.6)	21.4
Alpha-haemolytic *Streptococcus*	0 (0) ^3^	0 (0)	0	1 (14.3)	2 (28.6) ^5^	21.4
Methicillin-sensitive *Staphylococcus aureus*	0 (0)	1 (20)	0	0 (0)	1 (14.3) ^1,2^	7.1
*Haemophilus influenzae*	0 (0)	0 (0)	0	2 (28.6) ^7^	0 (0)	14.3
Total number of positive cultures	4	4	8	7	7	14

^1^ One patient (pre-PCV7) grew *Staphylococcus epidermidis* in the pleural fluid, but this was felt to be a contaminant; ^2^ The patient (post-PVC13) who grew *Staphylococcus aureus* grew it in a broncho-alveolar lavage culture, and it was felt to be pathogenic; ^3^ Two patients (pre-PCV7) grew Group A *Streptococcus* in a sputum culture; ^4^ One patient (post-PCV) had *Streptococcus pneumoniae* isolated from blood and pleural cultures; ^5^ Three patients (post-PCV13) grew Group A *Streptococcus* in a throat culture; ^6^ 12 patients (post-PCV13) had positive throat specimens for *Mycoplasma pneumoniae* (PCR or culture); ^7^ One patient had non-typeable *Haemophilus influenzae* (biotype II); type was not available for the other patient’s *Haemophilus influenzae*.

**Table 4:** Incidence of PCOMP and chest tube placements per 100,000 children, for children <4 years of age living in the CLHIN (by year).

Year	Period	At-Risk Population	Empyema	Parapneumonic Effusion	All Effusions	Chest Tube	Lung Abscess	Necrotizing Pneumonia	Total PCOMP Cases
2002	1	62,174	3.217	1.608	4.825	3.217	4.825	0.000	9.650
2003	1	62,027	4.837	4.837	9.673	6.449	0.000	0.000	9.673
2004	1	61,677	3.243	3.243	6.485	1.621	1.621	0.000	8.107
2005	2	61,061	6.551	6.551	13.102	8.189	1.638	0.000	14.739
2006	2	60,596	4.951	8.251	13.202	6.601	0.000	0.0000	13.202
2007	2	60,516	6.610	1.652	8.262	6.610	0.000	3.305	11.567
2008	2	60,700	9.885	6.590	16.475	4.942	0.000	0.000	16.475
2009	2	60,730	8.233	6.587	14.820	9.880	3.293	1.647	19.760
2010	2	60,812	9.866	11.511	21.377	9.866	0.000	0.000	21.377
2011	2	60,768	16.456	11.519	27.975	23.038	0.000	3.291	31.266
2012	3	60,434	8.274	8.274	16.547	8.274	0.000	0.0000	16.547
2013	3	60,107	4.991	8.318	13.310	8.318	0.000	1.664	14.973
2014	3	59,960	5.003	8.339	13.342	8.339	0.000	3.336	16.678
2015	3	59,806	3.344	6.688	10.032	5.016	1.672	1.672	13.377
*X*^2^ for overall trend ^1^			14.98	11.27	21.27	24.90	19.66	14.50	17.87
*p* for overall trend			0.31	0.59	0.068	0.024	0.10	0.34	0.16
*X*^2^ for pre-PCV-7 vs. post-PCV-13 ^2^			0.60	3.91	3.97	2.48	2.69	3.09	3.23
*p* for pre-PCV-7 vs. post-PCV-13			0.44	0.048	0.046	0.12	0.10	0.14 ^3^	0.072

Periods: (1): Pre-PCV7; (2): Post-PCV7 and pre-PCV13; (3): post-PCV13; ^1^ Degrees of freedom for chi-square with multiple groups = 13; ^2^ Degrees of freedom for chi-square = 1. ^3^ Fisher’s exact test.

**Table 5:** Incidence of PCOMP and chest tube placements per 100,000 children, for children four years of age and older living in the CLHIN (by year).

Year	Period	At-Risk Population	Empyema	Parapneumonic Effusion	All Effusions	Chest Tube	Lung Abscess	Necrotizing Pneumonia	Total PCOMP Cases
2002	1	217,611	1.379	0.919	2.298	1.379	0.000	0.000	2.298
2003	1	217,093	2.764	1.382	4.146	4.146	0.000	0.461	4.606
2004	1	215,869	0.926	0.463	1.390	0.463	0.926	0.000	2.316
2005	2	213,713	3.275	2.340	5.615	3.275	0.000	0.000	5.615
2006	2	212,088	2.829	2.829	5.658	4.244	0.472	0.472	6.601
2007	2	211,804	1.889	3.777	5.666	3.777	0.944	0.000	6.610
2008	2	212,448	2.824	0.941	3.766	2.354	0.000	0.000	3.766
2009	2	212,554	5.175	8.468	13.644	7.527	0.470	0.941	15.055
2010	2	212,843	2.819	5.638	8.457	3.759	0.940	0.470	9.866
2011	2	212,689	4.232	7.993	12.224	6.582	0.940	0.470	13.635
2012	3	211,518	4.728	6.146	10.874	7.092	0.000	0.473	11.347
2013	3	210,376	1.901	1.901	3.803	2.377	0.475	0.475	4.753
2014	3	209,861	2.859	8.101	10.960	5.718	0.000	0.953	11.913
2015	3	209,321	2.866	9.555	12.421	4.300	0.478	1.433	14.332
*X*^2^ for overall trend ^1^			13.84	67.43	65.55	30.02	11.33	12.00	70.65
*p* for overall trend			0.39	<0.001	<0.001	0.005	0.58	0.53	<0.001
*X*^2^ for pre-PCV-7 vs. post-PCV-13 ^2^			2.90	27.57	26.85	8.38	0.06	3.15	28.30
*p* for pre-PCV-7 vs. post-PCV-13			0.089	<0.001	<0.001	0.003	0.80	0.076	<0.001

Periods: (1): pre-PCV7; (2): post-PCV7 and pre-PCV13; (3): post-PCV13; ^1^ Degrees of freedom for chi-square with multiple groups = 13; ^2^ Degrees of freedom for chi-square = 1.

## Supplementary Materials

The following are available online at http://www.mdpi.com/2227-9067/5/3/36/s1, Figure S1: Admissions for PCOMP (all ages) for children living in Western Quebec, by year, Table S1: ICD codes used to identify patients with parapneumonic effusion, empyema, necrotizing pneumonia, and/or lung abscess.
