# Supplementary material for: Trends in Pediatric Complicated Pneumonia in an Ontario Local Health Integration Network
Source: Children (Basel). 2018 Mar 3;5(3):36. doi: 10.3390/children5030036 (PMC5867495; doi:10.3390/children5030036)
Supplement: Supplementary file 1 [file children-05-00036-s001.pdf]

Figure S1. Admissions for pediatric complicated pneumonia (PCOMP) (all ages) for children living in Western Quebec, by year.

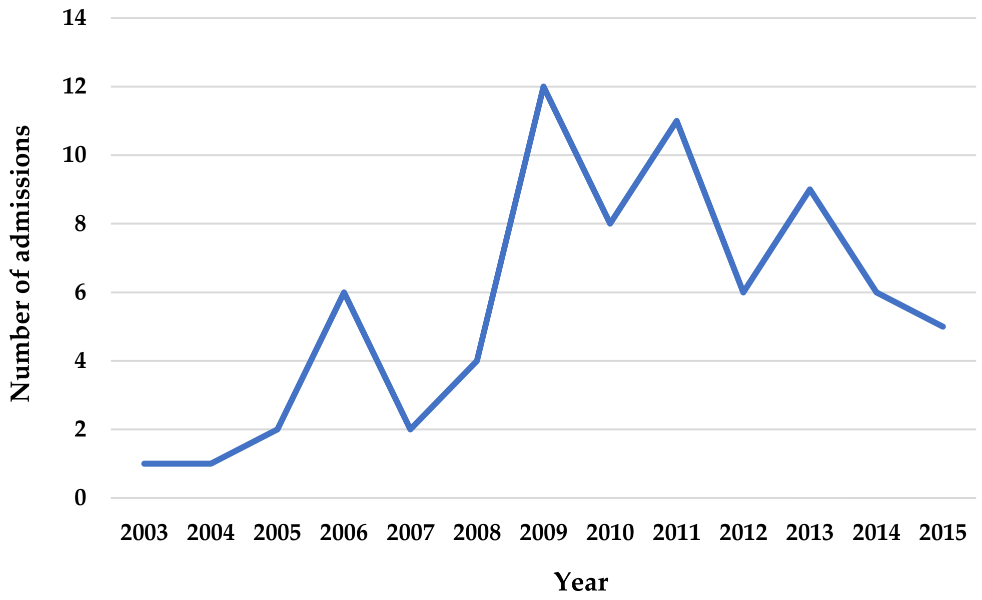

Supplementary Table S1. International Statistical Classification of Diseases and Related Health Problems (ICD) codes used to identify patients with parapneumonic effusion, empyema, necrotizing pneumonia, and/or lung abscess.

**Diagnosis Code**	**Description**
J10.0	Influenza with pneumonia, other influenza virus identified
J11.0	Influenza with pneumonia, virus not identified
J11.1	Influenza with other respiratory manifestations, virus not identified
J12.0	Adenoviral pneumonia
J12.1	Respiratory syncytial virus pneumonia
J12.2	Parainfluenza virus pneumonia
J12.3	Human metapneumovirus pneumonia*
J12.8	Other viral pneumonia
J12.9	Viral pneumonia, unspecified
J13	Pneumonia due to *Streptococcus pneumoniae*
J14	Pneumonia due to *Haemophilus influenzae*
J15.0	Pneumonia due to *Klebsiella pneumoniae*
J15.1	Pneumonia due to *Pseudomonas*
J15.2	Pneumonia due to *Staphylococcus*
J15.4	Pneumonia due to other streptococci
J15.5	Pneumonia due to *Escherichia coli*
J15.6	Pneumonia due to other Gram-negative bacteria
J15.7	Pneumonia due to *Mycoplasma pneumoniae*
J15.8	Other bacterial pneumonia
J15.9	Bacterial pneumonia, unspecified
J17.0	Pneumonia in bacterial diseases classified elsewhere
J17.1	Pneumonia in viral diseases classified elsewhere
J17.2	Pneumonia in mycoses
J18.0	Bronchopneumonia, unspecified
J18.1	Lobar pneumonia, unspecified
J18.9	Pneumonia, unspecified
J85.0	Gangrene and necrosis of lung
J85.1	Abscess of lung with pneumonia
J85.2	Abscess of lung without pneumonia
J85.3	Abscess of mediastinum
J86.0	Pyothorax with fistula
J86.9	Pyothorax without fistula
J90	Pleural effusion, not elsewhere classified
J91	Pleural effusion in conditions classified elsewhere
J94.0	Chylous effusion

*This code was introduced in 2009.
